# Application of CRISPR/Cas9-based mutant enrichment technique to improve the clinical sensitivity of plasma *EGFR* testing in patients with non-small cell lung cancer

**DOI:** 10.1186/s12935-022-02504-2

**Published:** 2022-02-15

**Authors:** Boyeon Kim, Yoonjung Kim, Saeam Shin, Seung-Tae Lee, Jae Yong Cho, Kyung-A. Lee

**Affiliations:** 1 Department of Laboratory Medicine, Gangnam Severance Hospital, Yonsei University College of Medicine, 211 Eonju-Ro, Gangnam-Gu, Seoul, 06273 Republic of Korea; 2 Department of Laboratory Medicine, Yonsei University College of Medicine, Seoul, 03722 Republic of Korea; 3 Division of Medical Oncology, Department of Internal Medicine, Gangnam Severance Hospital, Yonsei University College of Medicine, Seoul, 06273 Republic of Korea

**Keywords:** Cell-free nucleic acids, *EGFR* gene, Liquid biopsy, CRISPR-Cas System, Non-small cell lung cancer

## Abstract

**Background:**

Approximately 50%–60% of secondary resistance to primary *EGFR*- tyrosine kinase inhibitors (TKI) therapy is caused by acquired p.Thr790Met (T790M) mutation; however, highly fragmented, low-quantity circulating tumor DNA is an obstacle for detecting mutations. Therefore, more sensitive mutation detection techniques are required. Here, we report a new mutant enrichment technology, the CRISPR system combined with post-polymerase chain reaction (PCR) cell-free DNA (cfDNA) (CRISPR-CPPC) to detect the T790M mutation using droplet digital PCR (ddPCR) from cfDNA.

**Methods:**

The CRISPR-CPPC process comprises the following three steps: (1) cfDNA PCR, (2) assembly of post-PCR cfDNA and CRISPR/CRISPR associated protein 9 complex, and (3) enrichment of the target DNA template. After CRISPR-CPPC, the target DNA was detected using ddPCR. We optimized and validated CRISPR-CPPC using reference cfDNA standards and cfDNA from patients with non-small cell lung cancer who underwent TKI therapy. We then compared the detection sensitivity of CRISPR-CPPC assay with the results of real-time PCR and those of ddPCR.

**Results:**

CRISPR-CPPC aided detection of T790M with 93.9% sensitivity and 100% specificity. T790M mutant copies were sensitively detected achieving an approximately 13-fold increase in the detected allele frequency. Furthermore, positive rate of detecting a low T790M copy number (< 10 copies/mL) were 93.8% (15/16) and 43.8% (7/16) for CRISPR-CPPC assay and ddPCR, respectively.

**Conclusions:**

CRISPR-CPPC is a useful mutant enrichment tool for the sensitive detection of target mutation. When tested in patients with progressive disease, the diagnostic performance of CRISPR-CPPC assay is exceptionally better than that of any other currently available methods.

**Supplementary Information:**

The online version contains supplementary material available at 10.1186/s12935-022-02504-2.

## Background

Sensitive detection of epidermal growth factor receptor (*EGFR)* resistance mutation help to select third-generation *EGFR*- tyrosine kinase inhibitors (TKI) as a second-line treatment in non-small cell lung cancer (NSCLC) patients with progressive disease (PD) after being administered with first-line TKIs [1–3]. Approximately 50%–60% of secondary resistance to primary *EGFR*-TKI therapy is caused by acquired p.Thr790Met (T790M) mutation [4]. Several studies have reported *EGFR*-T790M as a secondary *EGFR* resistance mutation as well as a de novo mutation arising from pretreatment with TKIs [5, 6]. Generally, ≥ 10 copies/mL of T790M can be detected by currently available methods, but about 50% of patients have a low T790M copy number (< 10 copies/mL), making T790M difficult to detect [7]. Nevertheless, patients with a low T790M copy number (< 10 copies/mL) have a similar response to third-generation *EGFR*-TKIs as those with a higher T790M copy number (≥ 10 copies/mL) [8].

For patients with NSCLC, liquid biopsy for detecting circulating tumor DNA (ctDNA) has been most commonly implemented [9]. Real-time PCR (qPCR), such as FDA-approved Roche cobas® *EGFR* Mutation Test v2 (Roche Molecular Systems, Pleasanton, CA, USA), is widely used in the clinical setting because of its ease of use and relatively low cost. However, the test requires at least 100 copies/mL of specific *EGFR* mutants for the sensitive detection of mutations [10]. If the mutant allele frequency of *EGFR* mutants is below 0.1%, it can hardly be detected by qPCR [11]. Many researchers suggest that sensitive detection of cell-free DNA (cfDNA) mutations can be accomplished using droplet digital PCR (ddPCR) and next-generation sequencing (NGS) [12–14]; however, mutations with less than 0.1% allele frequency can be randomly detected using current techniques [15]. Furthermore, highly fragmented and low-quantity ctDNA, a high background of wild-type (WT) alleles, and the rapid clearance of cfDNA are obstacles for detecting especially low allele frequency mutations in cfDNA [16, 17]. Therefore, strategies to improve the detection capability of clinically significant mutant alleles with exceptionally low copy numbers among circulating nucleic acids are needed [13].

Clustered regularly interspaced short palindromic repeats (CRISPR)/CRISPR-associated protein 9 (Cas9) system has been introduced in the molecular diagnostic field to improve detection capability. Active CRISPR/Cas9 is a versatile and precise tool for gene editing and targeting [18]. In previous studies, CRISPR system was used to increase analytical sensitivity in two approaches for low-frequency mutant DNA detection. First of all, CRISPR system was used to enrich mutant DNAs by selectively cleaving non-target DNAs [19, 20]; however, application suggested by Gu et al. was applied only to glycine or proline codons owing to the presence of a protospacer adjacent motif (PAM) sequence [20]. Another approach of using CRISPR system is by specifically sorting out the target region by using a deactivated Cas9 with immunomagnetic separation [21]. The CRISPR/Cas9 could be a promising technology for detecting *EGFR* mutations presenting at a very low concentration; however, to our best knowledge, the detecting capability of previously described methods was not sufficient for applying to clinical cfDNA samples harboring low allele variants (< 10 copies/mL). Furthermore, those methods were validated only with a limited number of patient samples.

Building on advances in the CRISPR system, we propose a new mutant enrichment technique called CRISPR system combined with post-PCR cfDNA (CRISPR-CPPC). In this study, we validated and optimized CRISPR-CPPC assay to overcome the challenges of using cfDNA and demonstrated its efficacy in detecting a low copy number of T790M mutation in cfDNA of TKI-resistant patients.

## Methods

### Study design

**Fig. 1:**
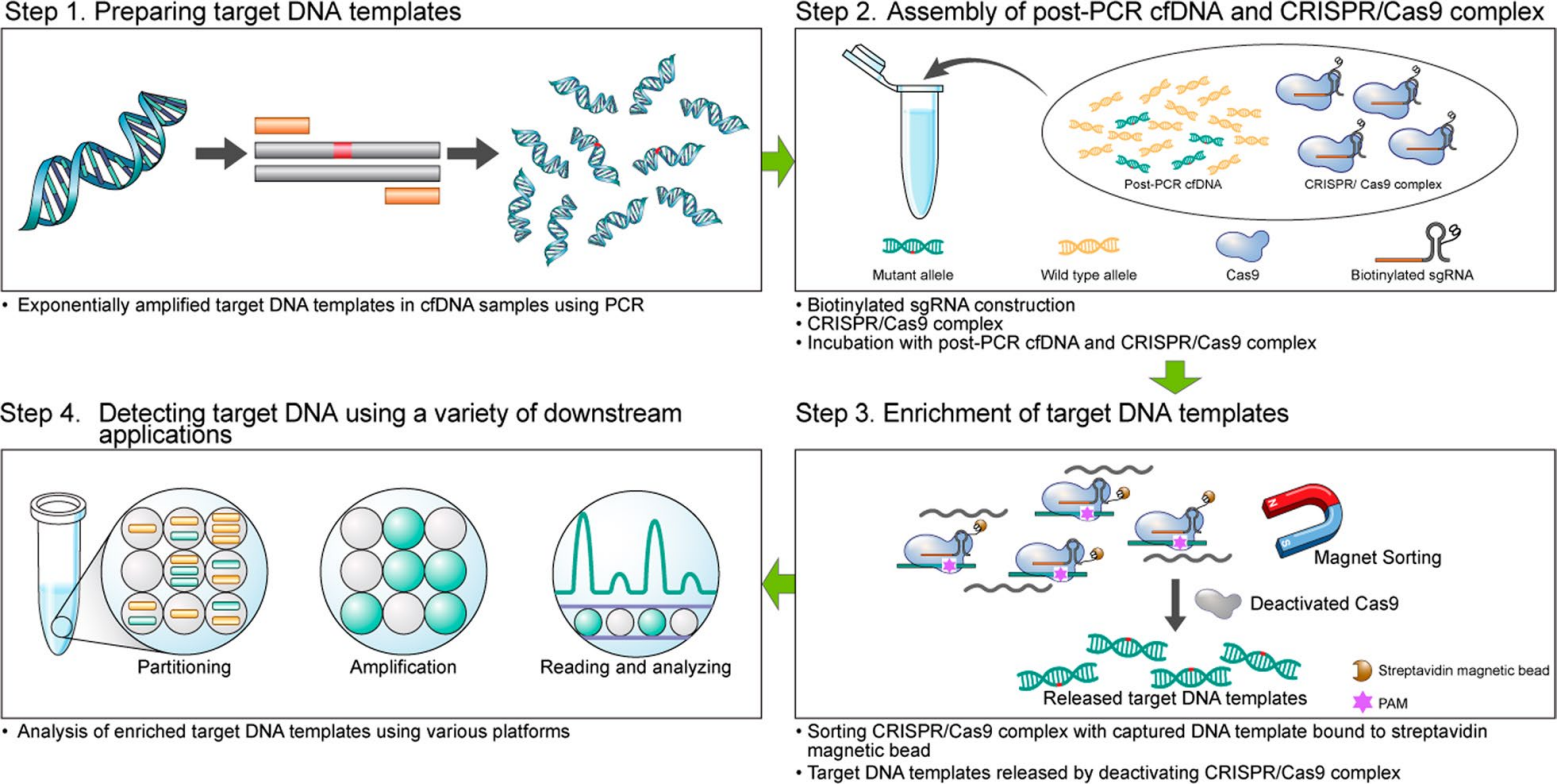
Schematic diagram of CRISPR-CPPC assay. CRISPR-CPPC comprises three steps: (1) cfDNA PCR, (2) assembly of post-PCR cfDNA and Cas9 complex, (3) enrichment of target DNA template. After CRISPR-CPPC, the target DNA can be detected using a variety of downstream applications, such as ddPCR

We developed a new mutant enrichment technology, CRISPR-CPPC, and optimized it to increase its diagnostic sensitivity. We validated CRISPR-CPPC assay with reference standards of mutant alleles and cfDNA from patients with NSCLC who had clinically progressed during or after *EGFR*-TKI treatment. The analytical performance of detecting *EGFR* T790M (NM_005228.4:c.2369C>T, p.Thr790Met) was evaluated by comparing the results of CRISPR-CPPC assay to those of qPCR or ddPCR. The study flowchart is shown in Additional file 1: Fig. S1. The CRISPR-CPPC comprises the following three steps: (1) cfDNA PCR, (2) assembly of post-PCR cfDNA and CRISPR/Cas9 complex, and (3) enrichment of the target DNA template. After CRISPR-CPPC, the target DNA can be detected using a variety of downstream applications. In this study, we used ddPCR as a downstream application for detecting T790M, and the nomenclature is established as follows: “ddPCR” means ddPCR without CRISPR-CPPC, and “CRISPR-CPPC assay” means CRISPR-CPPC analyzed using ddPCR. A schematic representation of CRISPR-CPPC and sgRNA target positions is shown in Fig. 1 and Additional file 1: Fig. S2.

### Patients

A total of 60 samples were collected from 51 patients who required *EGFR* gene mutation testing using Roche cobas® *EGFR* Mutation Test v2. The patients were admitted to two hospitals: Gangnam Severance Hospital and Severance Hospital located in Seoul, South Korea, from June 2018 to October 2020. Only patients with *EGFR*-mutated NSCLC who had clinically progressed during or after at least one first- or second-generation *EGFR*-TKI treatment cycle were included. Eight patients underwent one or two follow-up *EGFR* mutation tests. For all patients, *EGFR* genotyping was performed on the initial tissue biopsy obtained at the time of diagnosis. The study was approved by the Institutional Review Board of Gangnam Severance Hospital (IRB no. 3-2019-0393) and Severance Hospital (IRB no. 1-2019-0092). All patients provided written informed consent for specimen collection and genetic analysis. The need for the informed consent of the participants for reviewing medical records was waived on the condition that the research involves no more than minimal risk to the patients and their privacy.

### CRISPR-CPPC

#### Preparation of cfDNA

Blood samples were collected in vacutainer tubes containing EDTA or cfDNA collection tubes with a cell stabilizer, Cell-Free DNA BCT (Streck, La Vista, NE, USA). Blood samples were centrifuged at 1600×*g* for 10 min at 4 °C, followed by second high-spin centrifugation at 16,000×*g* for 10 min to separate the plasma from the peripheral blood cells. The plasma supernatant was stored at − 80 °C until cfDNA extraction. The MagMAX Cell-Free DNA Isolation Kit (Thermo Fisher Scientific, Waltham, MA, USA) was used to extract cfDNA. The concentration and size distribution of the nucleic acids were assessed using a 2200 TapeStation Instrument (Agilent Technologies, Santa Clara, CA, USA) with an Agilent High Sensitivity D1000 ScreenTape System (Agilent Technologies, Santa Clara, CA, USA).

#### cfDNA PCR

We designed the T790M primer sets for cfDNA PCR. Primer sequences are presented in Table 1. Cell-free DNA samples were processed by PCR before reacting with CRISPR/Cas9. PCR conditions were as follows: 95 °C for 5 min, followed by 35 cycles of 95 °C for 30 s, 62 °C for 30 s, and 72 °C for 1 min. The details of PCR effectiveness in CRISPR-CPPC are described in Additional file 1: Table S8 and Discussion.

**Table 1:** *EGFR* T790M primer information

sgRNA^*^	Forward	5′-TAATACGACTCACTATAGATCATGCAGCTCATGCCC-3′
	Reverse	5′-TTCTAGCTCTAAAACAAGGGCATGAGCTGCATGAT-3′
cfDNA PCR^†^	Forward	5′-CATGCGAAGCCACACTGAC-3′
	Reverse	5′-CGGACATAGTCCAGGAGGCA-3′

sgRNA, single guide RNA; cfDNA, cell-free DNA

^*^EGFR T790M primer for sgRNA

^†^Primer for cfDNA PCR. The expected product size was 164 bp

### Biotinylated sgRNA construction

The primer information for sgRNA is shown in Table 1. The sgRNA template was synthesized and purified using the GeneArt™ Precision gRNA Synthesis Kit (Thermo Fisher Scientific, Waltham, MA, USA) according to the manufacturer’s instructions, but we elongated the incubation time to 4 h for in vitro gRNA transcription. The yield of sgRNA was measured using the Qubit RNA BR Assay kit (Thermo Fisher Scientific, Waltham, MA, USA) to confirm that its yield was within the 10 to 40 µg range. The 3′-end of sgRNA was biotinylated using the Pierce™ RNA 3′-End Biotinylation Kit (Thermo Fisher Scientific, Waltham, MA, USA). The reactions were incubated overnight at 16 °C to increase efficiency.

#### CRISPR/Cas9 complex with post-PCR cfDNA

A CRISPR/Cas9 complex was constructed using biotinylated sgRNA, Cas9 nuclease, *Streptococcus pyogenes* (New England Biolabs, Ipswich, MA, USA), and the post PCR product of the cfDNA samples. Post PCR product was diluted to 50 ng of DNA according to manufacture’s instructions**.** The molar ratio of biotinylated sgRNA to Cas9 protein for a CRISPR/Cas9 complex was a 5:1 molar ratio in 20 µL, which led to a molar ratio of 1:400 of post-PCR cfDNA to Cas9 complex. The CRISPR/Cas9 complex and post-PCR cfDNA were incubated at 37 °C for 2 h in a thermocycler. Cas9 complexes trapping the target DNA were bound to the Dynabeads® MyOne™ Streptavidin C1 superparamagnetic beads (Thermo Fisher Scientific, Waltham, MA, USA) and released by heating to 65 °C.

### ddPCR assay

The number of T790M mutant copies in cfDNA samples before and after CRISPR-CPPC was quantified using ddPCR with the PrimePCR™ ddPCR™ Mutation Detection Assay kit (Bio-Rad, Hercules, CA, USA) according to the manufacturer’s instructions. Amplification was performed in a reaction volume of 20 µL using a QX100 Droplet Digital PCR System (Bio-Rad, Hercules, CA, USA). The PCR mix was composed of 10 µL of Bio-Rad Super mix TaqMan, 2 µL of T790M primer/probe mix, and 8 µL of post-CRISPR-CPPC cfDNA. The post-CRISPR-CPPC product was diluted 100-times for the optimal separation of false-positive and true-positive events. Thermal cycling conditions were as follows: 10 min at 95 °C, followed by 40 cycles of 95 °C for 30 s and 55 °C for 60 s. Results were analyzed with Quantasoft v.1.7.2 software (Bio-Rad, Hercules, CA, USA).

The methods of qPCR and NGS are written in Additional file 1.

### Data analysis

Quantification of the number of T790M mutant copies in the reaction was achieved by counting the number of positive and negative droplets. Event means absolute positive droplet count, but in this experiment, we started with 1 mL of plasma, therefore event can also be considered as copies/mL. When ddPCR was used for the samples that were not conditioned with CRISPR-CPPC, we considered positive if the measured events were ≥ 2 events/assay and negative if the events within a gated region were < 2 events/assay as described by Kim et al. [22].

However, when ddPCR was used for the samples conditioned with CRISPR-CPPC (CRISPR-CPPC assay), the limit of blank (LOB) and the limit of detection (LOD) were newly determined. LOB defined by the frequency of positive droplets measured in DNA-free samples conditioned with CRISPR-CPPC and the standard deviation of healthy controls were used to determine the LOD [22]. The LOD was determined as the lowest copy number concentration above the 95% confidence interval (CI) of the wild-type control conditioned with CRISPR-CPPC assay. The 95% CI was determined using the Poisson model and CLSI EP 17-A2 [23–26]. Based on the assessment of the LOB and the LOD, CRISPR-CPPC assays were considered positive if the measured events were ≥ 6 events/assay and negative if the events within a gated region were < 6 events/assay (Additional file 1: Table S1).

### Validation of CRISPR-CPPC assay

Before using CRISPR-CPPC assay for patient cfDNA samples, the method was validated using Multiplex I cfDNA Reference Standards (Horizon Discovery, Cambridge, United Kingdom), which included wild-type cfDNA with mutant allele frequencies of 5%, 1%, and 0.1%.

### Statistical analysis

Statistical analysis was performed using Microsoft Excel 2016 (Microsoft Corporation, Redmond, WA, USA). Overall percent agreement (OPA), negative percent agreement (NPA), and positive percent agreement (PPA) were calculated as described in the CLSI guidelines[27]. Data are presented using 95% CIs and two-sided *P-*values. Statistical significance was set at *P* ≤ 0.05.

## Results

### Patient characteristics

The characteristics of patients with *EGFR*-mutated NSCLC who had clinically progressed after *EGFR*-TKI treatment are described in Table 2. The median age was 62 years (range, 39–83 years), and thirty-six patients (70.6%) were females. Forty-three out of fifty-one patients had stage IV disease (84.3%). Thirty patients (58.8%) had exon 19 deletion, eighteen patients (35.3%) had L858R point mutation, two patients (3.9%) had S768I point mutation, one patient (2.0%) had L861Q point mutation, and one patient (2.0%) had G719S point mutation. Ten patients (19.6%) received erlotinib therapy, thirteen (25.5%) received afatinib, and twenty-seven (52.9%) received gefitinib therapy. One patient (2.0%) received gefitinib and erlotinib therapy at different time points. The median months from the start of TKI to the sample collection for *EGFR* testing were 17.5 months (range, 2–72 months).

**Table 2:** Patient characteristics

Characteristics	No. of Patients
	N = 51 (100%)
Age, median (range), years	62 (39–83)
Sex	
Female	36 (70.6%)
Male	15 (29.4%)
Histologic type	
Adenocarcinoma	50 (98.0%)
Squamous cell carcinoma	1 (2.0%)
Tumor stage	
IB	2 (3.9%)
IIIA	3 (5.9%)
IIIB	3 (5.9%)
IVA	21 (41.2%)
IVB	22 (43.1%)
M category*	
M1a	13 (25.5%)
M1b	10 (19.6%)
M1c	27 (52.9%)
M1a + M1c^†^	1 (2.0%)
Tissue *EGFR* genotyping	
Exon 19 deletion	29 (56.9%)
L858R	17 (33.3%)
S768I	2 (3.9%)
L861Q	1 (2.0%)
G719S	1 (2.0%)
Exon 19 deletion + L858R^‡^	1 (2.0%)
Previous *EGFR*-TKI therapy	
Erlotinib	10 (19.6%)
Afatinib	13 (25.5%)
Gefitinib	27 (52.9%)
> 1 *EGFR*-TKIs	1 (2.0%)

TKI, tyrosine kinase inhibitor

^*^ The information of M category was reclassified at the time of *EGFR* testing. M category was based on the 8th TMN edition. M1a: lung metastases or pleural/pericardial malignant effusion or nodules; M1b: a single metastatic lesion in a single distant organ; M1c: multiple lesions in a single organ or multiple lesions in multiple organs

^†^The M category of patient G (Table S7) was M1a at first *EGFR* testing. M stage was reclassified to M1c at second *EGFR* testing

^‡^1 patient had both exon 19 deletion and L858R

### Validation of CRISPR-CPPC assay

The analytical sensitivity of CRISPR-CPPC assay was evaluated using the Multiplex I cfDNA Reference Standard with allele frequencies of 5%, 1%, and 0.1% (Horizon Discovery, Cambridge, United Kingdom). The expected copy number of mutant alleles (3–109 copies) and the actual copy number of mutant alleles observed in these samples are presented in Table 3. The positive detection of mutant DNA after CRISPR-CPPC was approximately 2–6 times higher than the expected copies of mutant DNA. After mutant enrichment, the allele frequency was approximately 1.6–3.7 times higher than the expected allele frequency.

**Table 3:** Analytical sensitivity of CRISPR-CPPC assay in detecting *EGFR* T790M mutation

Reference Materials (T790M)	Expected allele frequency (%)^*^	Expected copies of mutant DNA per sample^*^	Expected copies of wild-type DNA per sample^*^	CRISPR-CPPC assay Detection positive (≥ 6 events/assay)		
				Observed Mutant allele frequency (%)	Copies of mutant DNA per sample	Copies of wild-type DNA per sample
5% Multiplex I cfDNA Reference Standard (HD777), 20 ng/µL	4.9	109	2120	8.8	231	2409
1% Multiplex I cfDNA Reference Standard (HD778), 20 ng/µL	1.1	24	2256	1.7	60	3376
0.1% Multiplex I cfDNA Reference Standard (HD779), 20 ng/µL	0.1	3	2228	0.5	19	3842

cfDNA, cell-free DNA; CRISPR-CPPC, CRISPR system combined with post-PCR cfDNA; ddPCR, droplet digital PCR

^*^ Expected allele frequency and copy number of wild-type and mutant DNA measured using ddPCR were provided by the manufacturer

### A comparison of qPCR, ddPCR, and CRISPR-CPPC assay

Sixty samples from fifty-one patients were analyzed. All samples were subjected to qPCR, ddPCR, and CRISPR-CPPC assay for detecting T790M (Additional file 1: Table S2). Samples that tested positive for T790M through two or more of the experimental methods (qPCR from cfDNA, tissue or other types of samples, NGS, ddPCR, and CRISPR-CPPC) were considered to be true positives (Additional file 1: Table S3). Based on the results of multiple assays, the sensitivities of CRISPR-CPPC assay and ddPCR were 92.0% and 64.0%, respectively (Additional file 1: Table S3). The PPA (%), NPA (%), and OPA (%) of CRISPR-CPPC assay and ddPCR compared to the qPCR results are presented in Additional file 1: Table S4. Compared to qPCR, CRISPR-CPPC assay and ddPCR showed 100% and 75% PPA, respectively. CRISPR-CPPC assay detected T790M variants from 15 samples whose T790M mutations were not detected by qPCR (Additional file 1: Table S4). When compared to ddPCR, the PPA (%), NPA (%), and OPA (%) was 88.2%, 62.8 and 70.0%, respectively (Additional file 1: Table S5). Eighteen samples showed discordant results between CRISPR-CPPC assay and ddPCR (Additional file 1: Table S6). CRISPR-CPPC assay detected T790M mutant alleles in sixteen T790M-negative samples by ddPCR, and ddPCR detected T790M in two T790M-negative samples by CRISPR-CPPC assay (sample No. 12 & 47) (Additional file 1: Tables S5, S6). These two samples were also tested by NGS, which showed that one sample was T790M-positive with an allele frequency of 0.2% and the other was T790M-negative. The final clinical diagnosis of clinical progression was made by oncologists based on the integration of patients' medical history and radiological findings. The researchers retrospectively reviewed the participant's medical records, including the final clinical diagnosis. Table 4 presents the analytical performance of CRIPSR-CPPC assay and ddPCR based on the results of multiple assays and final clinical diagnoses. The sensitivity and specificity of CRISPR-CPPC assay were increased up to 93.9% and 100.0%, respectively.

**Table 4:** Analytical performance of assays for detecting T790M mutation based on clinical diagnosis

Method	T790M mutation was confirmed with multiple studies and or/and clinical diagnosis^*^			Sensitivity (95% CI)	Specificity (95% CI)	Accuracy (95% CI)
	Results	Pos (n = 33)	Neg (n = 27)			
ddPCR	Pos	16	1	48.5% (30.8%–66.5%)	96.3% (81.0%–99.9%)	70.0% (56.8%–81.2%)
	Neg	17	26			
CRISPR-CPPC assay	Pos	31	0	93.9% (79.8%–99.3%)	100.0% (87.2%–100.0%)	96.7% (88.5%–99.6%)
	Neg	2	27			

Pos, positive; Neg, negative; CI, confidence interval; CRISPR-CPPC, CRISPR system combined with post-PCR cfDNA; ddPCR, droplet digital PCR; qPCR, real-time PCR; NGS, next-generation sequencing

^*^T790M detected by more than two methods (qPCR from cfDNA, tissue or other types of samples, NGS, ddPCR, CRISPR-CPPC assay, Clinical diagnosis) simultaneously is considered “true positive”. “Clinical diagnosis-T790M-positive” was defined when clinical history and image interpretation supported that a positive T790M result would be close to a true positive. Image interpretation was performed only for CRISPR-CPPC-positive samples

### Ultra-Sensitive Detection of CRISPR-CPPC assay

A comparison of the allele frequency and positive events of sixty samples is shown in Additional file 1: Table S2. Most samples showed approximately 1.2–13-times higher allele frequencies with the use of CRISPR-CPPC assay. In addition, approximately 1.6–562-times more positive events were detected with the use of CRISPR-CPPC assay. The copy number comparison between pairs was statistically significant, with a *P*-value of < 0.0001, using the Wilcoxon signed-rank test.

**Fig. 2:**
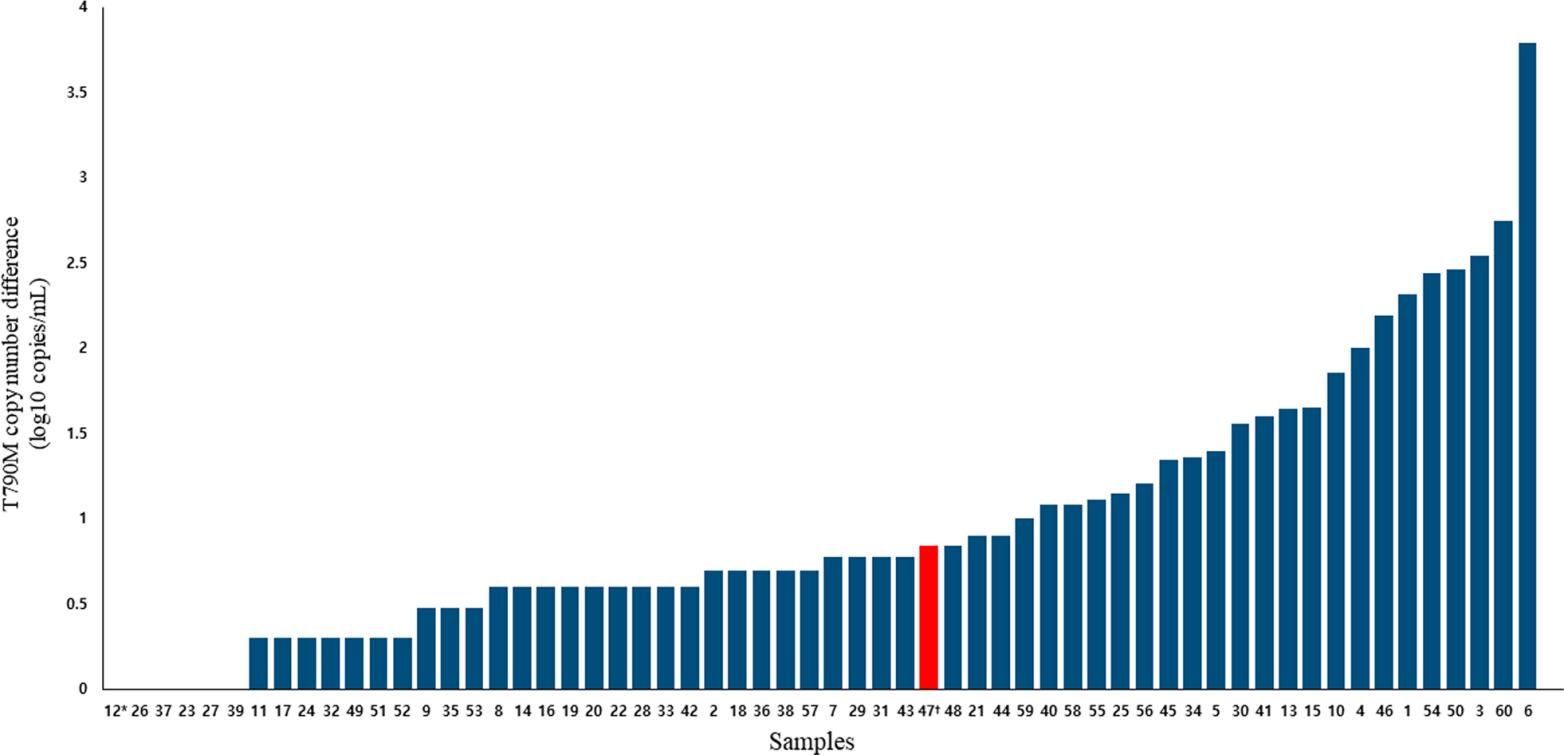
T790M-positive copy number differences between CRISPR-CPPC assay and ddPCR in all 60 samples: CRISPR-CPPC increased the T790M-positive copy numbers, except in sample number 47 (Additional file 1: Table S2). Seven samples with a copy difference of < 1 were not expressed on the log10-scaled y-axis

We evaluated the performance of CRISPR-CPPC assay using the samples containing low copies of T790M mutant alleles from patients with *EGFR*-mutated NSCLC who had clinically progressed after *EGFR*-TKI treatment. The distribution of T790M copies according to detecting assays was depicted in Additional file 1: Fig. S3. The overall T790M positive copy number differences between CRISPR-CPPC assay and ddPCR are shown in Fig. 2. Figure 2 shows the trend of CRISPR-CPPC assay increasing the T790M positive copy numbers compared to ddPCR, except for sample number 47. Among 51 samples with ≤ 10 copies of T790M alleles based on ddPCR, the positive T790M rate of ddPCR and CRISPR-CPPC assay was 15.7% (n = 8 / 51) and 45.1% (n = 23 / 51), respectively (Additional file 1: Fig. S3 and Table S2). When CRISPR-CPPC assay was tested in < 10 copies/mL of “true positive” T790M cfDNA samples, positive rate was 93.8% (15/16) compared to that of ddPCR (43.8% (7/16)) (Table 5). CRISPR-CPPC assay showed improved sensitivity of detecting T790M, notably in samples with T790M-low copies or T790M-negative by ddPCR.

**Table 5:** Data of < 10 copies/mL of true positive T790M patient samples

Sample no.	cfDNA qPCR	T790M ddPCR Detection positive (≥ 2 events/assay)				T790M CRISPR-CPPC assay Detection positive (≥ 6 events/assay)				Tissue or other samples qPCR	NGS
	Result	Events	Wild	Allele frequency (%)	Result	Events	Wild	Allele frequency (%)	Result	Result	Result
3	ND	3	560	0.5	Positive	353	4570	7.2	Positive	L858R	NA
4	Positive	1	238	0.4	ND	102	1811	5.3	Positive	Exon19 del	T790M 2.4%
7	Positive	0	533	0.0	ND	6	1228	0.5	Positive	L858R	NA
8	Positive	3	430	0.7	Positive	7	1556	0.4	Positive	Exon19 del	NA
9	Positive	5	438	1.1	Positive	8	1215	0.7	Positive	Exon19 del	NA
29	ND	3	1170	0.3	Positive	9	2568	0.3	Positive	Exon19 del	NA
30	ND	2	386	0.5	Positive	38	2766	1.4	Positive	L858R	NA
32	ND	0	302	0.0	ND	2	1978	0.1	ND	Exon19 del T790M from lung tissue	T790M 0.5%
34	ND	1	310	0.3	ND	24	3816	0.6	Positive	Exon19 del T790M from lung tissue	NA
45	Positive	6	205	2.8	Positive	28	1220	2.2	Positive	Exon19 del	NA
46	Positive	2	114	1.7	Positive	158	7316	2.1	Positive	Exon19 del	NA
48	ND	0	71	0.0	ND	7	2119	0.3	Positive	Exon19 del	T790M 0.1%
54	Positive	0	31	0.0	ND	274	3874	6.6	Positive	L858R	NA
58	ND	0	139	0.0	ND	12	6052	0.2	Positive	Exon19 del	T790M 0.2%
59	ND	0	11	0.0	ND	10	5294	0.2	Positive	L858R T790M from pleural fluid	ND
60	Positive	1	137	0.7	ND	562	6343	8.1	Positive	Exon19 del	T790M 2.1%

cfDNA, cell-free DNA; CRISPR-CPPC, CRISPR system combined post-PCR cfDNA; ddPCR, droplet digital PCR; NA, not applicable; ND, not detected; qPCR, real-time PCR; SQI, semiquantitative index

^*^T790M detected by more than two methods (qPCR from cfDNA,tissue or other types of samples, NGS, ddPCR, CRISPR-CPPC assay) simultaneously is considered “true positive”

### The monitoring *EGFR* T790M in patient samples using CRISPR-CPPC assay

Among the 51 patients, eight patients had one or two follow-up *EGFR* mutation tests using the Roche cobas® *EGFR* Mutation Test v2. As shown in Additional file 1: Table S7, patients E, G, and H had a follow-up test to detect T790M using CRISPR-CPPC assay, but qPCR was unable to detect T790M. Using ddPCR, 0, 3, and 0 positive events with a respective allele frequency (%) of 0, 0.3, and 0 were detected. Using CRISPR-CPPC assay in the first sample from patient H, the T790M variant was detected with six positive events with an allele frequency (%) of 0.1. In the second sample from patient E and patient G, T790M was detected with eight and nine positive events and an allele frequency (%) of 0.2 and 0.3, respectively. These results indicate that only CRISPR-CPPC assay could detect exceptionally low copies of T790M in patient samples.

## Discussion

In the routine process of treating patients with NSCLC, a great deal of effort is devoted to detecting *EGFR* mutations. Of late, clinical trials for many third-generation TKIs are underway [28, 29]. As the application of the mutation detection of cfDNA increases and becomes important in the field of precision medicine, the need for more sensitive mutation detection remains. A mutant enrichment technique combined with a sensitive detection tool is a feasible solution. Kim et al. reported the enrichment method using exosomal total nucleic acids (exoTNA) which harbored more abundant target mutant alleles to improve the analytical sensitivity of detecting *EGFR* mutation in the extraction step. However, the positive rate of short-length exoTNA was only 50.0% (N = 5/10) in cases with low T790M copies(< 10 copies/mL) [22]. We considered the CRISPR/Cas9 system as a suitable method for the application to enrich low mutant alleles after the extraction step. Thus, CRISPR-CPPC assay could be used with not just cfDNA but also with exosomal nucleic acids.

CRISPR/Cas9 system has been developed and applied to detect low-frequency mutant DNA [19–21]. However, previous methods had a problem with DNA template loss during the process of mutation enrichment. For example, immunomagnetic capture could lead to loss of template resulting in T790M detection rates (%) of only 50% and 42.8% at AF of 1% and 0.1% samples, respectively [21]. Therefore, we developed and validated CRISPR-CPPC assay that could enrich mutant alleles after the extraction step to increase the diagnostic sensitivity in cases with low T790M copies (< 10 copies/mL). To overcome the limitation caused by the characteristics of cfDNA when using CRISPR/Cas9 in clinical samples in previous studies [21, 30], we added “PCR step” in CRISPR-CPPC. As a result, CRISPR-CPPC assay demonstrated 93.8% (15/16) of positive rate for detecting from low copies of T790M (< 10 copies/mL) (Table 5), indicating that CRISPR/Cas9 could capture a very low abundance of mutant DNA in the extremely high background of non-mutant DNA.

Furthermore, the previous methods were only validated with limited number of patient cfDNA samples [19–21]. We evaluated the diagnostic performance of CRISPR-CPPC assay by comparing results of qPCR, ddPCR, and CRISPR-CPPC assay tested in 60 cfDNA samples of patients with NSCLC. When “True T790M positive” is considered to be simultaneous T790M detection by more than two methods (qPCR from cfDNA, tissue or other types of samples, NGS, ddPCR, CRISPR-CPPC assay), the sensitivity and specificity of CRISPR-CPPC assay was 92.0% and 77.1%, respectively. Compared to the sensitivity and specificity of ddPCR (64.0% and 97.1%), CRISPR-CPPC assay demonstrated higher sensitivity but lower specificity. However, even though CRISPR-CPPC assay is a more powerful detection tool than other current methods, if the true positivity criteria are too stringent, CRISPR-CPPC assay will appear as if it gives false-positive results. If so, even though the analytical performance of CRISPR-CPPC assay is superior to other comparable methods, its superiority may not be evident. Therefore, with consideration of clinically meaningful effects of T790M, we added clinical evaluation criteria to minimize false negatives for evaluating the clinical performance of CRISPR-CPPC assay. We used clinical history and image interpretation information under the supervision of an oncologist to provide additional evidence of "Clinical diagnosis-T790M-positive” in Table 4. When we defined “Clinical diagnosis-T790M-positive”, CRISPR-CPPC assay can detect T790M with 93.9% sensitivity and 100% specificity (Table 4).

One case (sample number 32) was shown to be T790M-negative by both ddPCR and CRISPR-CPPC assay, but T790M-positive with an allele frequency of 0.2% by NGS. In this case, CRISPR-CPPC assay could not have detected T790M because mutant copies have not been amplified due to the extremely little amount of DNA input (< 0.1 ng) for the PCR step (Additional file 1: Table S6). NGS was conducted using a fresh sample but both ddPCR and CRISPR-CPPC assay used cfDNA samples extracted from the stored plasma, which might have been degraded.

CRISPR-CPPC has demonstrated several advantages: First, it is easy to use as long as the target primer is designed. Second, it can successfully enrich samples with a low number of mutant copies (< 10 copies/mL). Third, it can clarify the results in samples that previously had borderline results. Fourth, based on the experiment, enrichment reactions can be performed with the same amount (approximately 0.4 ng) of post-PCR cfDNA, indicating that CRISPR-CPPC can become a standardized process. Finally, CRISPR-CPPC compensated for DNA template loss by adding the PCR step for cfDNA; however, this compensation step for DNA loss could lead to possible contamination, therefore, careful handling is required.

This study has several limitations. We hybridized CRISPR-CPPC with ddPCR because ddPCR was a rapid and highly sensitive method for detecting variants. We optimized dilution factor for post-CRISPR-CPPC product to apply to ddPCR platform. When applying to other platforms (qPCR, NGS, etc.), further optimizing process should be required. CRISPR-CPPC requires a PAM sequence to assemble the CRISPR/Cas9 complex; however, the PAM sequence (5′-NGG-3′) can be found on average 8–12 bp in the human genome [31–33]. Therefore, this would not greatly hamper the application of CRISPR-CPPC to the human genome. Finally, CRISPR-CPPC cannot be used for patient monitoring because its quantitative application has not yet been evaluated. Therefore, the results of CRISPR-CPPC should only be considered qualitatively. Although this approach met the study’s original purpose of enriching low mutant copies to render them detectable, it needs to be developed as a quantitative tool to be used for both diagnostic and monitoring patient care purposes. Incorporating dead Cas9 into CRISPR-CPPC may be able to help with resolving this problem [34] by reducing loss of mutant allele during heat elution stage, but further study is required.

This study shows that the performance of CRISPR-CPPC assay is exceptionally better than that of any other currently available methods and that it can be easily used in clinical settings. Therefore, CRISPR-CPPC can be clinically applied to facilitate gene expression profiling, diagnosis, and the selection of appropriate treatment regimens.

## Conclusions

In conclusion, the proposed CRISPR-CPPC technology is a useful mutant enrichment tool for the sensitive detection of target mutations. CRISPR-CPPC enriches the mutations by adding PCR step and using CRISPR/Cas9 to recognize the target without cleaving the target or non-target. We demonstrated the capability of CRISPR-CPPC assay to detect low copy number mutations in the cfDNA of patients with TKI resistance, possibly caused by the T790M mutation, which is undetectable in the patients by current FDA-approved methods. Thus, CRISPR-CPPC assay can greatly contribute to diagnosis and the selection of appropriate treatment regimens by facilitating the sensitive detection of mutations in cfDNA.

## Supplementary Information

**Additional file 1.** Additional Methods: The methods of qPCR and NGS assay. Additional Discussion: Evaluation of PCR step in CRISPR-CPPC using cfDNA from patients with NSCLC. Table S1: LOB and LOD of CRISPR-CPPC assay for T790M mutation. Table S2: Application of CRISPR-CPPC assay on patient samples. Table S3: Analytical performance of assays for detecting T790M mutation in cfDNA from patients with NSCLC presenting disease progression on 1st or 2nd generation EGFR-TKI. Table S4: Comparison of test results of qPCR to those of CRISPR-CPPC assay and ddPCR for EGFR T790M in cell-free plasma DNA. Table S5: Comparison of the test results of ddPCR to those of CRISPR-CPPC assay for EGFR T790M in cell-free plasma DNA. Table S6: Cases with different ddPCR and CRISPR-CPPC assay results. Table S7: Application of CRISPR-CPPC assay on follow-up patient samples. Table S8: Evaluation of PCR step in CRISPR-CPPC using cfDNA from patients with NSCLC. Fig S1: Study flow chart. Fig S2: Schematic illustration of the CRISPR target site around the human EGFR T790 locus. Fig S3: Distribution of T790M copies from sixty samples in this study.

## Data Availability

The data that support the findings of this study, contain clinical outcomes for which IRB requires approval prior to analysis. Therefore, the data are not publicly available. The data will be made available to authorized researchers who have obtained institutional review board (IRB) approval from their own institution and from Gangnam Severance Hospital, Yonsei University, Seoul, Republic of Korea IRB. For data access requests, please contact the corresponding author, Dr. Kyung-A Lee, email address: kal1119@yuhs.ac.

## Abbreviations

Cas9: CRISPR associated protein 9
cfDNA: Cell-free DNA
CI: Confidence interval
ctDNA: Circulating tumor DNA
CRISPR-CPPC: CRISPR system combined post-PCR cfDNA
ddPCR: Droplet digital PCR
EGFR: Epidermal growth factor receptor
exoTNA: exosomal total nucleic acids
LOB: Limit of blank
LOD: Limit of detection
NGS: Next-generation sequencing
NPA: Negative percent agreement
NSCLC: Non-small cell lung cancer
OPA: Overall percent agreement
PAM: Protospacer-adjacent motif
PCR: Polymerase chain reaction
PD: Progressive disease
PPA: Positive percent agreement
qPCR: Real-time PCR
sgRNA: Single guide RNA
TKI: Tyrosine kinase inhibitor
WT: Wild-type
