# Supplementary material for: Application of CRISPR/Cas9-based mutant enrichment technique to improve the clinical sensitivity of plasma EGFR testing in patients with non-small cell lung cancer
Source: Cancer Cell Int. 2022 Feb 15;22:82. doi: 10.1186/s12935-022-02504-2 (PMC8845274; doi:10.1186/s12935-022-02504-2)
Supplement: Supplementary file 1 — Additional file 1. Additional Methods: The methods of qPCR and NGS assay. Additional Discussion: Evaluation of PCR step in CRISPR-CPPC using cfDNA from patients with NSCLC. Table S1: LOB and LOD of CRISPR-CPPC assay for T790M mutation. Table S2: Application of CRISPR-CPPC assay on patient samples. Table S3: Analytical performance of assays for detecting T790M mutation in cfDNA from patients with NSCLC presenting disease progression on 1st or 2nd generation EGFR-TKI. Table S4: Comparison of test results of qPCR to those of CRISPR-CPPC assay and ddPCR for EGFR T790M in cell-free plasma DNA. Table S5: Comparison of the test results of ddPCR to those of CRISPR-CPPC assay for EGFR T790M in cell-free plasma DNA. Table S6: Cases with different ddPCR and CRISPR-CPPC assay results. Table S7: Application of CRISPR-CPPC assay on follow-up patient samples. Table S8: Evaluation of PCR step in CRISPR-CPPC using cfDNA from patients with NSCLC. Fig S1: Study flow chart. Fig S2: Schematic illustration of the CRISPR target site around the human EGFR T790 locus. Fig S3: Distribution of T790M copies from sixty samples in this study. [file 12935_2022_2504_MOESM1_ESM.docx]

**Additional Information**

**Additional Methods:** The methods of qPCR and NGS assay

**Additional Discussion**: Evaluation of PCR step in CRISP-CPPC using cfDNA from patients with NSCLC

**Additional Tables**

Table S1: LOB and LOD of CRISPR-CPPC assay for T790M mutation

Table S2: Application of CRISPR-CPPC assay on patient samples

Table S3: Analytical performance of assays for detecting T790M mutation in cfDNA from patients with NSCLC presenting disease progression on 1st or 2nd generation EGFR-TKI

Table S4: Comparison of test results of qPCR to those of CRISPR-CPPC assay and ddPCR for EGFR T790M in cell-free plasma DNA

Table S5: Comparison of the test results of ddPCR to those of CRISPR-CPPC assay for EGFR T790M in cell-free plasma DNA

Table S6: Cases with different ddPCR and CRISPR-CPPC assay results

Table S7: Application of CRISPR-CPPC assay on follow-up patient samples

Table S8: Evaluation of PCR step in CRISPR-CPPC using cfDNA from patients with NSCLC

**Additional Figures**

Fig S1: Study flow chart

Fig S2: Schematic illustration of the CRISPR target site around the human *EGFR* T790 locus

Fig S3: Distribution of T790M copies from sixty samples in this study

**Additional Methods:** The methods of qPCR and NGS assay

**Roche cobas^®^ *EGFR* Mutation Test v2 (qPCR assay)**

The qPCR assay was executed using cobas^®^ *EGFR* Mutation Test v2 according to manufacturer’s instructions. 75 uL of DNA from each plasma sample (2 mL) was loaded into three reaction wells (25 uL DNA per well). Amplification and detection were performed using the Cobas z 480 analyzer (Roche Molecular Systems, Inc., Branchburg, NJ, USA). The data were interpreted using the Cobas z 480 software.

**NGS assay**

The NGS results are referenced from the NGS report using either Grardant360 CDx (Guardant Health, Redwood City, CA, USA) or Oncomine Pan-Cancer Cell Free Assay (Thermo Fisher Scientific). Patient samples from Severance Hospital were sent to a laboratory in the United States where Guardant360 CDx (Guardant Health, Redwood City, CA, USA) was used, following the manufacturer’s instructions. Patient samples from Gangnam Severance Hospital was prepared using the Oncomine Pan-Cancer Cell-Free Assay (Thermo Fisher Scientific). The NGS preparation using Oncomine Pan-Cancer Cell-Free Assay followed the Kim et al.’s method [1]. The libraries were prepared using >5 ng input of nucleci acid according to manufacturer’s instructions. Templating and sequencing were performed using the Ion 540^TM^ Kit on the Ion Chef^TM^ and on the Ion S5 XL system (Thermo Fisher Scientific). The alignment to the hg19 human reference genome and variant calling were executed using Torrent Suite^TM^ and Ion Reporter^TM^ software version 5.10, respectively. The molecular coverage depth and read coverage depth at the target base were provided by the Torrent SuiteTM software. The average of median reac coverage and median molecular coverage were 36,095 x and 1934 x, respectively. Variants with an allele frequency of >0.1% were reported, and the measured allele frequency (%) was calculated from the division of mutant coverage depth by the total coverage depth.

**Additional Discussion**: Evaluation of PCR step in CRISPR-CPPC using cfDNA from patients with NSCLC

We tested the CRISPR/Cas9 without PCR step as other known study has done [2]. When there were not PCR step before applying CRISPR/Cas9, there were lack of reproducibility of applying CRISPR/Cas9 for the mutant enrichment in our experiment (data not shown). We contemplated that this lack of reproducibility may be caused by the characteristics of cfDNA such as highly fragmented and low-quantity, and the loss of DNA during the sorting step [2]. Usually in clinical settings, cfDNA is used to detect EGFR mutations, but the limitations of previous methods are drawbacks for deriving the reliable results. Therefore, to get reliable results in liquid biopsy samples, we thought PCR step is necessary as a part of mutant enrichment. So, we tried adding PCR step to compensate the disadvantages of previous study. With the aid of PCR step, CRISPR/Cas9 could capture a very low abundance of mutant DNA in the extremely high background of non-mutant DNA (Table S8). And we proved that without CRISPR/Cas9, only simply adding the PCR step in ddPCR had failed to detect T790M mutant alleles since it would also amplify extremely high background of wild-type alleles (Table S8).

1. Kim Y, Shin S, Lee KA. Exosome-based detection of EGFR T790M in plasma and pleural fluid of prospectively enrolled non-small cell lung cancer patients after first-line tyrosine kinase inhibitor therapy. Cancer Cell Int 2021;21:50.

2. Aalipour A, Dudley JC, Park SM, Murty S, Chabon JJ, Boyle EA, et al. Deactivated CRISPR Associated Protein 9 for Minor-Allele Enrichment in Cell-Free DNA. Clin Chem 2018;64:307-16.

**Table S1.** LOB and LOD of CRISPR-CPPC assay for T790M mutation

| Number of samples | Blank^*^ | T790M (-)  Healthy control cfDNA^†^ |  |
| --- | --- | --- | --- |
|  | Positive events | Positive events |  |
| 1 | 0 | 1 |  |
| 2 | 0 | 0 |  |
| 3 | 0 | 2 |  |
| 4 | 0 | 2 |  |
| 5 | 0 | 0 |  |
| 6 | 0 | 1 |  |
| 7 | 0 | 1 |  |
| 8 | 0 | 5 |  |
| 9 | 0 | 2 |  |
| 10 | 0 | 2 |  |
| 11 | 0 | 5 |  |
| Event | 0 | 21 |  |
| Mean | 0 | 1.9 |  |
| SD | 0 | 1.6 |  |
| LOB | 0 |  |  |
| LOD (CLSI EP17-A2) |  | 3 |  |
| 95% CI upper bound (one-tail Poisson distribution) |  | 2.5 |  |
| ^*^Analysis of CRISPR-CPPC assay blank samples without DNA (N = 11) | | | |
| ^†^11 healthy subjects were anonymized and studied as control samples | | | |
| Abbreviations: CI, confidence interval; LOB, limit of blank; LOD, limit of detection; SD, standard deviation | | | |

**Table S2.** Application of CRISPR-CPPC assay on patient samples

| Sample No. | T790M qPCR^‡^ | T790M ddPCR  Detection positive  (≥2 events/assay) | | | | T790M CRISPR-CPPC assay Detection positive  (≥6 events/assay) | | | |
| --- | --- | --- | --- | --- | --- | --- | --- | --- | --- |
|  | Result | Events | Wild | Allele frequency (%) | Result | Events | Wild | Allele frequency (%) | Result |
| 1 | Positive | 17 | 293 | 5.5 | Positive | 224 | 1257 | 15.1 | Positive |
| 2 | ND | 0 | 423 | 0.0 | ND | 5 | 2336 | 0.2 | ND |
| 3 | ND | 3 | 560 | 0.5 | Positive | 353 | 4570 | 7.2 | Positive |
| 4 | Positive | 1 | 238 | 0.4 | ND | 102 | 1811 | 5.3 | Positive |
| 5 | Positive | 22 | 423 | 4.9 | Positive | 47 | 2727 | 1.7 | Positive |
| 6 | Positive | 758 | 1864 | 28.9 | Positive | 6932 | 12176 | 36.3 | Positive |
| 7 | Positive | 0 | 533 | 0.0 | ND | 6 | 1228 | 0.5 | Positive |
| 8 | Positive | 3 | 430 | 0.7 | Positive | 7 | 1556 | 0.4 | Positive |
| 9 | Positive | 5 | 438 | 1.1 | Positive | 8 | 1215 | 0.7 | Positive |
| 10 | Positive | 16 | 362 | 4.2 | Positive | 88 | 2333 | 3.6 | Positive |
| 11 | ND | 1 | 191 | 0.5 | ND | 3 | 3138 | 0.1 | ND |
| 12^*^ | ND | 3 | 194 | 1.5 | Positive | 3 | 2579 | 0.1 | ND |
| 13 | Positive | 18 | 2068 | 0.9 | Positive | 62 | 2402 | 2.5 | Positive |
| 14 | ND | 0 | 648 | 0.0 | ND | 4 | 2536 | 0.2 | ND |
| 15 | Positive | 42 | 1221 | 3.3 | Positive | 87 | 807 | 9.7 | Positive |
| 16 | ND | 0 | 32 | 0.0 | ND | 4 | 683 | 0.6 | ND |
| 17 | ND | 0 | 401 | 0.0 | ND | 2 | 1870 | 0.1 | ND |
| 18 | ND | 0 | 590 | 0.0 | ND | 5 | 2071 | 0.2 | ND |
| 19 | ND | 0 | 602 | 0.0 | ND | 4 | 3796 | 0.1 | ND |
| 20 | ND | 0 | 146 | 0.0 | ND | 4 | 3036 | 0.1 | ND |
| 21 | ND | 0 | 412 | 0.0 | ND | 8 | 3589 | 0.2 | Positive |
| 22 | ND | 0 | 1886 | 0.0 | ND | 4 | 872 | 0.5 | ND |
| 23 | ND | 0 | 5416 | 0.0 | ND | 1 | 1181 | 0.1 | ND |
| 24 | ND | 0 | 292 | 0.0 | ND | 2 | 3114 | 0.1 | ND |
| 25 | ND | 0 | 481 | 0.0 | ND | 14 | 2776 | 0.5 | Positive |
| 26 | ND | 0 | 513 | 0.0 | ND | 0 | 1180 | 0.0 | ND |
| 27 | ND | 0 | 39 | 0.0 | ND | 1 | 736 | 0.1 | ND |
| 28 | ND | 0 | 29 | 0.0 | ND | 4 | 1012 | 0.4 | ND |
| 29 | ND | 3 | 1170 | 0.3 | Positive | 9 | 2568 | 0.3 | Positive |
| 30 | ND | 2 | 386 | 0.5 | Positive | 38 | 2766 | 1.4 | Positive |
| 31 | ND | 0 | 162 | 0.0 | ND | 6 | 3995 | 0.1 | Positive |
| 32 | ND | 0 | 302 | 0.0 | ND | 2 | 1978 | 0.1 | ND |
| 33 | ND | 0 | 542 | 0.0 | ND | 4 | 4108 | 0.1 | ND |
| 34 | ND | 1 | 310 | 0.3 | ND | 24 | 3816 | 0.6 | Positive |
| 35 | ND | 1 | 2432 | 0.0 | ND | 4 | 2013 | 0.2 | ND |
| 36 | ND | 0 | 1111 | 0.0 | ND | 5 | 4803 | 0.1 | ND |
| 37 | ND | 0 | 134 | 0.0 | ND | 0 | 1326 | 0.0 | ND |
| 38 | ND | 0 | 155 | 0.0 | ND | 5 | 2460 | 0.2 | ND |
| 39 | ND | 1 | 207 | 0.5 | ND | 2 | 1440 | 0.1 | ND |
| 40 | ND | 0 | 172 | 0.0 | ND | 12 | 1394 | 0.9 | Positive |
| 41 | Positive | 51 | 721 | 6.6 | Positive | 91 | 912 | 9.1 | Positive |
| 42 | ND | 0 | 601 | 0.0 | ND | 4 | 8345 | 0.0 | ND |
| 43 | ND | 0 | 110 | 0.0 | ND | 6 | 5712 | 0.1 | Positive |
| 44 | ND | 0 | 1541 | 0.0 | ND | 8 | 9580 | 0.1 | Positive |
| 45 | Positive | 6 | 205 | 2.8 | Positive | 28 | 1220 | 2.2 | Positive |
| 46 | Positive | 2 | 114 | 1.7 | Positive | 158 | 7316 | 2.1 | Positive |
| 47^†^ | ND | 12 | 410 | 2.8 | Positive | 5 | 4709 | 0.1 | ND |
| 48 | ND | 0 | 71 | 0.0 | ND | 7 | 2119 | 0.3 | Positive |
| 49 | ND | 0 | 98 | 0.0 | ND | 2 | 3618 | 0.1 | ND |
| 50 | Positive | 28 | 762 | 3.5 | Positive | 319 | 3713 | 7.9 | Positive |
| 51 | ND | 0 | 112 | 0.0 | ND | 2 | 726 | 0.3 | ND |
| 52 | ND | 0 | 44 | 0.0 | ND | 2 | 2870 | 0.1 | ND |
| 53 | ND | 0 | 44 | 0.0 | ND | 3 | 4128 | 0.1 | ND |
| 54 | Positive | 0 | 31 | 0.0 | ND | 274 | 3874 | 6.6 | Positive |
| 55 | ND | 0 | 63 | 0.0 | ND | 13 | 7324 | 0.2 | Positive |
| 56 | ND | 0 | 201 | 0.0 | ND | 16 | 7243 | 0.2 | Positive |
| 57 | ND | 0 | 19 | 0.0 | ND | 5 | 3641 | 0.1 | ND |
| 58 | ND | 0 | 139 | 0.0 | ND | 12 | 6052 | 0.2 | Positive |
| 59 | ND | 0 | 11 | 0.0 | ND | 10 | 5294 | 0.2 | Positive |
| 60 | Positive | 1 | 137 | 0.7 | ND | 562 | 6343 | 8.1 | Positive |

* T790M was negative by next-generation sequencing (NGS)

^†^ T790M was positive with an allele frequency of 0.2% by NGS

^‡^T790M was detected using Roche cobas® *EGFR* Mutation Test v2

Abbreviations: CRISPR-CPPC, CRISPR system combined post-PCR cfDNA; ddPCR, droplet digital PCR; ND, not detected; qPCR, real-time PCR; SQI, semiquantitative index

**Table S3.** Analytical performance of assays for detecting T790M mutation in cfDNA from patients with NSCLC presenting disease progression on 1^st^ or 2^nd^ generation *EGFR*-TKI

| Method | T790M mutation was confirmed with multiple assays^*^ | | | Sensitivity (95%CI) | Specificity (95%CI) | Accuracy (95%CI) |
| --- | --- | --- | --- | --- | --- | --- |
|  | Results | Pos (n=25) | Neg (n=35) |  |  |  |
| ddPCR | Pos | 16 | 1 | 64.0% ( 42.5% to 82.0%) | 97.1% ( 85.1% to 99.9%) | 83.3% (71.5% to 91.7%) |
|  | Neg | 9 | 34 |  |  |  |
| CRISPR-CPPC assay | Pos | 23 | 8 | 92.0% (74.0% to 99.0%) | 77.1% (59.9% to 89.6%) | 83.3% (71.5% to 91.7%) |
|  | Neg | 2 | 27 |  |  |  |

^*^T790M detected by more than two methods (qPCR from cfDNA, tissue or other types of samples, NGS, ddPCR, CRISPR-CPPC) simultaneously is considered “true positive”.

Abbreviations: NSCLC, non-small cell lung cancer; *EGFR*-TKI, epidermal growth factor receptor-tyrosine kinase inhibitor; Pos, positive; Neg, negative; CI, confidence interval; CRISPR-CPPC, CRISPR system combined post-PCR cfDNA; ddPCR, droplet digital PCR; qPCR, real-time PCR; NGS, next-generation sequencing

**Table S4.** Comparison of test results of qPCR to those of CRISPR-CPPC assay and ddPCR for *EGFR* T790M in cell-free plasma DNA

|  |  | qPCR | |  |  | |  |
| --- | --- | --- | --- | --- | --- | --- | --- |
|  |  | Positive | Negative |  | |  | |
| CRISPR-CPPC assay | Positive | 16 | 15 | PPA (%) (95% CI) | | 100.0 (80.6 – 100) | |
|  | Negative | 0 | 29 | NPA (%) (95% CI) | | 65.9 (51.1 – 78.1) | |
|  |  |  |  | OPA (%) (95% CI) | | 75.0 (62.8 – 84.2) | |
| ddPCR | Positive | 12 | 5 | PPA (%) (95% CI) | | 75.0 (50.5 – 89.8) | |
|  | Negative | 4 | 39 | NPA (%) (95% CI) | | 88.6 (76.0 – 95.0) | |
|  |  |  |  | OPA (%) (95% CI) | | 85.0 (73.9 – 91.9) | |

Abbreviations: CI, confidence interval; CRISPR-CPPC, CRISPR system combined with post-PCR cfDNA; ddPCR, droplet digital polymerase chain reaction; NPA, negative percent agreement; OPA, overall percent agreement; PPA, positive percent agreement; qPCR, real-time PCR

**Table S5.** Comparison of the test results of ddPCR to those of CRISPR-CPPC assay for *EGFR* T790M in cell-free plasma DNA

|  |  | ddPCR | |  |  |  |  |  |
| --- | --- | --- | --- | --- | --- | --- | --- | --- |
|  |  | Positive | Negative |  | |  | |  |
| CRISPR-CPPC assay | Positive | 15 | 16 | PPA (%) (95% CI) | | 88.2 (65.7 – 96.7) | |  |
|  | Negative | 2^*^ | 27 | NPA (%) (95% CI) | | 62.8 (47.9 – 75.6) | |  |
|  |  |  |  | OPA (%) (95% CI) | | 70.0 (57.5 – 80.1) | |  |

^*^ NGS was performed, and it was confirmed that one sample was T790M-positive with an allele frequency of 0.2% and the other sample was T790M-negative.

Abbreviations: CI, confidence Interval; CRISPR-CPPC, CRISPR system combined with post-PCR cfDNA; ddPCR, droplet digital PCR; NPA, negative percent agreement; OPA, overall percent agreement; PPA, positive percent agreement

**Table S6.** Cases with different ddPCR and CRISPR-CPPC assay results

| Sample No. | Sex | Age | First- or second-generation *EGFR*-TKIs | Tissue Genotyping at diagnosis | DNA input (ng) | Plasma collection date (Months since TKI) | CRISPR-CPPC | | | | qPCR (SQI) | ddPCR | | NGS | Tissue genotyping at disease progression | Image (CT, MRI, PET-CT) interpretation at disease progression^*^ |
| --- | --- | --- | --- | --- | --- | --- | --- | --- | --- | --- | --- | --- | --- | --- | --- | --- |
|  |  |  |  |  |  |  | Positive event (≥6 events/assay) | | Wild event | AF (%) |  | Positive event (≥2 events/assay) | |  |  |  |
| CRISPR-CPPC assay-Positive | | | | | | | | | | | | | | | | |
| 4 | M | 55 | Afatinib | Exon19 deletion | 0.96 | 14 | Pos | 102 | 1811 | 5.3 | Pos (8.45) | Neg | 1 | T790M 2.4% | N/T |  |
| 7 | M | 77 | Gefitinib | L858R | 0.58 | 7 | Pos | 6 | 1228 | 0.5 | Pos (4.00) | Neg | 0 | Not tested | N/T |  |
| 21^*^ | F | 69 | Afatinib | Exon19 deletion | 1.41 | 8 | Pos | 8 | 3589 | 0.2 | Neg | Neg | 0 | Not tested | N/T | ^*^Hepatic metz R/O bone metz |
| 25^*^ | F | 59 | Afatinib | L858R | 1.07 | 12 | Pos | 14 | 2776 | 0.5 | Neg | Neg | 0 | Not tested | N/T | ^*^New metz at right adrenal gland R/O malignant pleural effusion |
| 31^*^ | F | 75 | Gefitinib | L858R | 0.58 | 18 | Pos | 6 | 3995 | 0.1 | Neg | Neg | 0 | Not tested | N/T | ^*^Aggravation of lung cancer Pleural seeding metz with malignant effusion Metz in both lungs |
| 34 | F | 85 | Gefitinib | Exon19 deletion | 1.28 | 16 | Pos | 24 | 3816 | 0.6 | Neg | Neg | 1 | Not tested | T790M (+) |  |
| 40^*^ | F | 68 | Gefitinib | Exon19 deletion | 0.71 | 17 | Pos | 12 | 1394 | 0.9 | Neg | Neg | 0 | Not tested | N/T | ^*^Pleural seeding at metastatic lesions |
| 43^*†^ | M | 43 | Gefitinib | Exon19 deletion | 2.26 | 46 | Pos | 6 | 5712 | 0.1 | Neg | Neg | 0 | Not tested | N/T | ^*^PD Brain metz |
| 44^*†^ | M | 44 | Gefitinib | Exon19 deletion | 14.5 | 48 | Pos | 8 | 9580 | 0.1 | Neg | Neg | 0 | Not tested | N/T | ^*^PD |
| 48 | F | 59 | Gefitinib | Exon19 deletion | 1.66 | 14 | Pos | 7 | 2119 | 0.3 | Neg | Neg | 0 | T790M 0.1% | N/T |  |
| 54 | F | 72 | Gefitinib | L858R | <0.1 | 31 | Pos | 274 | 3874 | 6.6 | Pos (4.99) | Neg | 0 | Not tested | N/T |  |
| 55^*^ | M | 59 | Gefitinib | L858R | <0.1 | 9 | Pos | 13 | 7324 | 0.2 | Neg | Neg | 0 | Not tested | N/T | ^*^R/O metz at brain Diffused metz in entire both lungs |
| 56^*^ | F | 70 | Gefitinib | G719S | 1.38 | 13 | Pos | 16 | 7243 | 0.2 | Neg | Neg | 0 | Not tested | N/T | ^*^PD Endobronchial invasion and pleural seeding metz Left pleural metz with pleural effusion.  Increased metastatic lesion in the left temporal lobe of brain. |
| 58 | F | 46 | Afatinib | Exon19 deletion | <0.1 | 8 | Pos | 12 | 6052 | 0.2 | Neg | Neg | 0 | T790M 0.2% | N/T |  |
| 59 | F | 49 | Afatinib | L858R | <0.1 | 54 | Pos | 10 | 5294 | 0.2 | Neg | Neg | 0 | Neg | T790M (+) |  |
| 60 | F | 60 | Afatinib | Exon19 deletion | 0.68 | 11 | Pos | 562 | 6343 | 8.1 | Pos (8.36) | Neg | 1 | T790M 2.1% | N/T |  |
| CRISPR-CPPC assay-Negative | | | | | | | | | | | | | | | | |
| 12 | F | 61 | Gefitinib | L858R | 1.09 | 24 | Neg | 3 | 2579 | 0.1 | Neg | Pos | 3 | Neg | N/T |  |
| 47 | M | 57 | Gefitinib | Exon19 deletion | <0.1 | 6 | Neg | 5 | 4709 | 0.1 | Neg | Pos | 12 | T790M 0.2% | N/T |  |

^*^ Clinical history and image interpretation support that a positive CRISPR-CPPC assay T790M result would be close to a true positive.

^†^ Same patient. The second test was performed three months after the first test.

Abbreviations: AF, allele frequency; CRISPR-CPPC, CRISPR system combined post-PCR cfDNA; CT, computed tomography; ddPCR, droplet digital PCR; metz, metastasis; MRI, magnetic resonance imaging; NGS, next-generation sequencing; N/T, not tested; PD, progressive disease; PET-CT, positron emission tomography-computed tomography; Pos, positive; qPCR, real-time PCR; R/O, rule out; SQI, semiquantitative index; TKI, tyrosine kinase inhibitor.

**Table S7.** Application of CRISPR-CPPC assay on follow-up patient samples

|  | T790M qPCR | T790M ddPCR | | | T790M CRISPR-CPPC assay | | |
| --- | --- | --- | --- | --- | --- | --- | --- |
|  | SQI* | Events | Wild | Allele frequency (%) | Events | Wild | Allele frequency (%) |
| Patient A | ND | 1 | 191 | 0.5 | 3 | 3138 | 0.1 |
| Patient A | ND | 3 | 194 | 1.5 | 3 | 2579 | 0.1 |
| Patient A | 9.29 | 18 | 2068 | 0.9 | 62 | 2402 | 2.5 |
| Patient B | ND | 0 | 648 | 0.0 | 4 | 2536 | 0.2 |
| Patient B | 10.74 | 42 | 1221 | 3.3 | 87 | 807 | 9.7 |
| Patient C | ND | 0 | 32 | 0.0 | 4 | 683 | 0.6 |
| Patient C | ND | 0 | 401 | 0.0 | 2 | 1870 | 0.1 |
| Patient D | ND | 0 | 590 | 0.0 | 5 | 2071 | 0.2 |
| Patient D | ND | 0 | 602 | 0.0 | 4 | 3796 | 0.1 |
| Patient E | ND | 0 | 146 | 0.0 | 4 | 3036 | 0.1 |
| Patient E | ND | 0 | 412 | 0.0 | 8 | 3589 | 0.2 |
| Patient F | ND | 0 | 1886 | 0.0 | 4 | 872 | 0.5 |
| Patient F | ND | 0 | 5416 | 0.0 | 1 | 1181 | 0.1 |
| Patient G | ND | 0 | 29 | 0.0 | 4 | 1012 | 0.4 |
| Patient G | ND | 3 | 1170 | 0.3 | 9 | 2568 | 0.3 |
| Patient H | ND | 0 | 110 | 0.0 | 6 | 5712 | 0.1 |
| Patient H | ND | 0 | 1541 | 0.0 | 8 | 9580 | 0.1 |
| * SQI was measured with Roche cobas^®^ *EGFR* Mutation Test v2 | | | | | |  |  |
| Abbreviations: CRISPR-CPPC, CRISPR system combined post-PCR cfDNA; ddPCR, droplet digital PCR; ND, not detected; qPCR, real-time PCR; SQI, semiquantitative index | | | | | | | |

**Table S8.** Evaluation of PCR step in CRISPR-CPPC using cfDNA from patients with NSCLC

| Sample No. | T790M qPCR^1^ | T790M ddPCR Detection positive  (≥2 events/assay) | | | | T790M ddPCR with post-PCR product  Detection positive  (≥2 events/assay) | | | | T790M ddPCR with 1000x diluted post-PCR product Detection positive  (≥2 events/assay) | | | | T790M CRISPR-CPPC assay Detection positive  (≥6 events/assay) | | | |
| --- | --- | --- | --- | --- | --- | --- | --- | --- | --- | --- | --- | --- | --- | --- | --- | --- | --- |
|  | Result | Events | Wild | Allele frequency (%) | Result | Events | Wild | Allele frequency (%) | Result | Events | Wild | Allele frequency (%) | Result | Events | Wild | Allele frequency (%) | Result |
| 7 | Positive | 0 | 533 | 0.0 | ND | 0 | 19140 | 0.0 | ND | 1 | 16483 | 0.0 | ND | 6 | 1228 | 0.5 | Positive |
| 34 | ND | 1 | 310 | 0.3 | ND | 0 | 17735 | 0.0 | ND | 0 | 16426 | 0.0 | ND | 24 | 3816 | 0.6 | Positive |
| 54 | Positive | 0 | 31 | 0.0 | ND | 0 | 14852 | 0.0 | ND | 0 | 17624 | 0.0 | ND | 274 | 3874 | 6.6 | Positive |
| 60 | Positive | 1 | 137 | 0.7 | ND | 0 | 15181 | 0.0 | ND | 0 | 18986 | 0.0 | ND | 562 | 6343 | 8.1 | Positive |
| ^1^T790M was detected using Roche cobas® EGFR Mutation Test v2  Abbreviations: CRISPR-CPPC, CRISPR system combined post-PCR cfDNA; ddPCR, droplet digital PCR; ND, not detected; qPCR, real-time PCR; SQI, semiquantitative index | | | | | | | | | | | | | | | | |  |


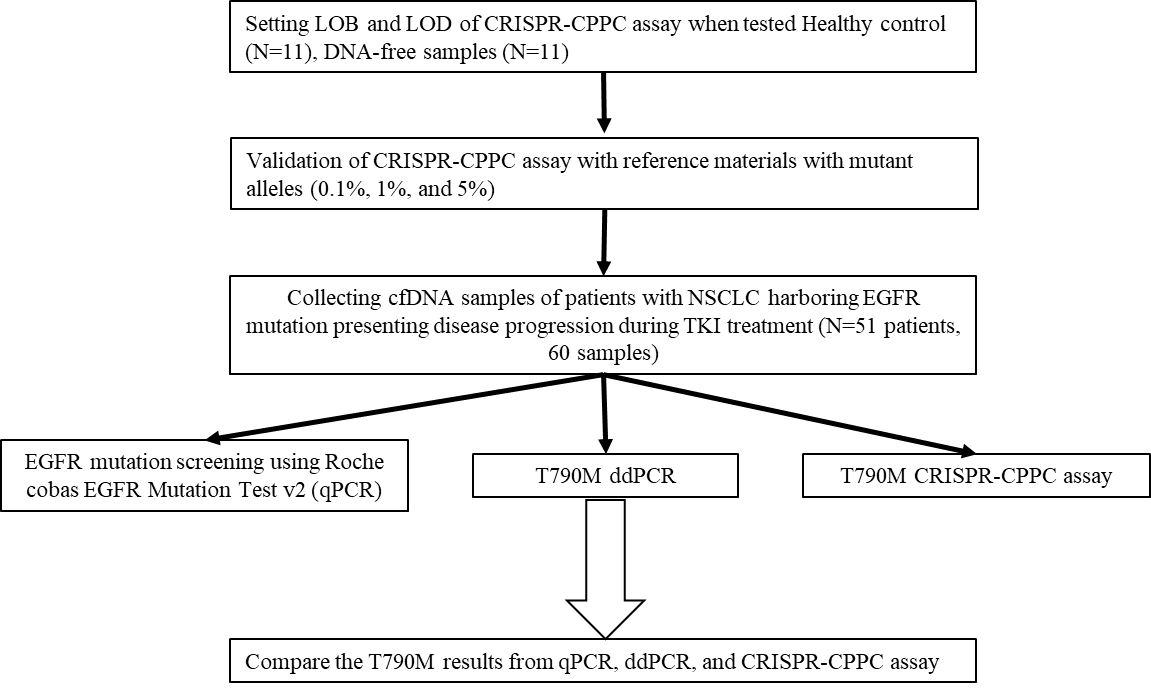


Validation of CRISPR-CPPC assay with reference materials with mutant alleles (0.1%, 1%, and 5%)

Setting LOB and LOD of CRISPR-CPPC assay when tested Healthy control (N=11), DNA-free samples (N=11)

**Fig S1.** Study Flow chart


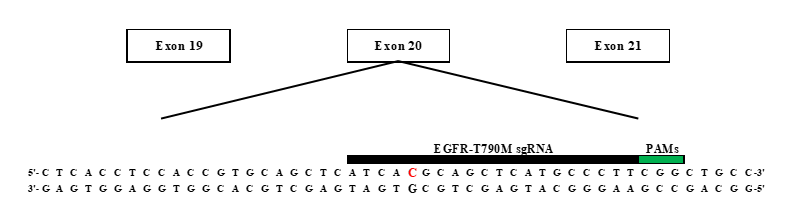


**Fig S2.** Schematic illustration of the CRISPR target site around the human *EGFR* T790 locus


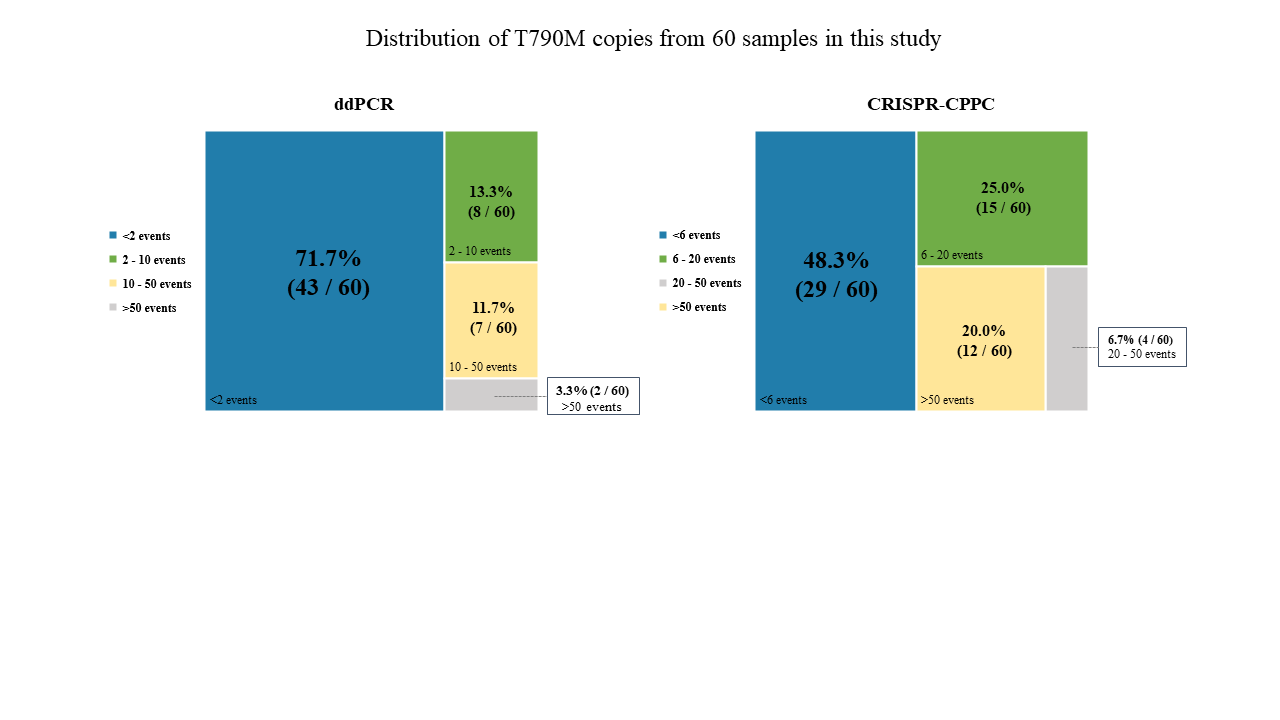


**Fig S3.** Distribution of T790M copies from sixty samples in this study
